# Self-oxygenating PCN-224(Mn) co-delivers sorafenib and plumbagin to boost chemo-photodynamic therapy in hepatocellular carcinoma

**DOI:** 10.1016/j.ijpx.2026.100554

**Published:** 2026-04-28

**Authors:** Xiang Wang, Hengrui Li, Le Wang, Yihan Ma, Miao Qin, Ruonan Sun, Jing Hu

**Affiliations:** a Wuxi School of Medicine, Jiangnan University, Wuxi 214122, China; b School of Biotechnology and Key Laboratory of Carbohydrate Chemistry and Biotechnology of Ministry of Education, Jiangnan University, Wuxi 214122, China; c School of Food Science and Technology, Jiangnan University, Wuxi 214122, China; d Institute of Future Food Technology, JITRI, No.19 Wenzhuang Road, Yixing 214200, China

**Keywords:** Hepatocellular carcinoma, PCN-224(Mn), Photodynamic therapy, Self‑oxygenation, Chemotherapy

## Abstract

Tumor hypoxia is a major barrier to oxygen-dependent photodynamic therapy (PDT), markedly limiting its therapeutic efficacy in hepatocellular carcinoma (HCC). Herein, a self‑oxygenating and redox-amplifying metal-organic framework (MOF) nanoplatform is constructed by co-loading sorafenib (Sor) and plumbagin (PLB) into a surface galactose functionalized Mn-porphyrinic PCN-224(Mn) (PM) framework to afford SorPLB@Gal-PM for asialoglycoprotein receptor (ASGPR)-mediated HCC targeting. The Mn-porphyrin centers endow SorPLB@Gal-PM with catalase-like activity, enabling *in situ* decomposition of endogenous H_2_O_2_ to generate O_2_ and thereby sustaining ^1^O_2_-based PDT under irradiation. Meanwhile, Sor/PLB co-delivery, together with PLB-mediated GPX4 suppression, promotes oxidative stress amplification. In hypoxic liver cancer stem cell-like C5WN1 cells, SorPLB@Gal-PM elicits markedly enhanced intracellular ROS upon irradiation, showing approximately 3.4-fold and 2.9-fold higher DCFH-DA signals than SorPLB@Gal-PCN-224 and PM, respectively, accompanied by downregulation of hypoxia-inducible factor-1α (HIF-1α) and glutathione peroxidase 4 (GPX4). In a C5WN1 tumor-bearing nude mouse model, SorPLB@Gal-PM combined with irradiation achieves the most potent tumor suppression with a tumor inhibition rate of 83.70% ± 4.12%. Collectively, SorPLB@Gal-PM provides a hypoxia-adaptive nanoplatform integrating Mn-assisted self‑oxygenation and redox-amplified chemo-PDT for effective HCC therapy.

## Graphical abstract

**Figure:**
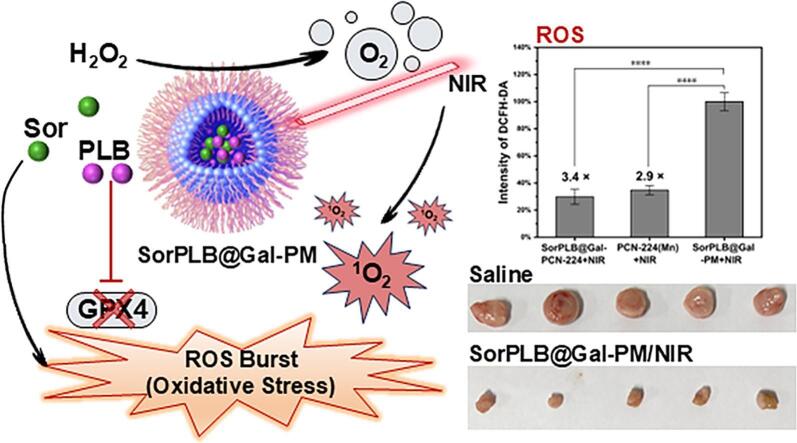

## 1 Introduction

Liver cancer is a leading cause of cancer-related death worldwide, and hepatocellular carcinoma (HCC) accounts for the majority of cases (Zhou et al., 2025; Hwang et al., 2025). Despite advances in systemic therapies, the therapeutic outcome of HCC remains unsatisfactory, largely owing to the complex tumor microenvironment (TME) (Zheng et al., 2025). Among the key TME features, hypoxia is particularly pervasive in solid tumors because of aberrant vasculature and insufficient perfusion (Bae et al., 2024). Hypoxia triggers the activation of hypoxia-inducible factor-1α (HIF-1α) in tumor cells, which drives malignant progression and is closely associated with treatment resistance, thereby posing a major barrier to effective antitumor therapy (Wicks and Semenza, 2022; Ladd et al., 2024; Moris et al., 2025).

Photodynamic therapy (PDT) is attractive due to its spatiotemporal controllability and minimal invasiveness (Ailioaie et al., 2025; Zhang et al., 2023a; Zhang et al., 2025; Ji et al., 2022; Shuai et al., 2026). However, conventional type II PDT depends on molecular oxygen to generate singlet oxygen (^1^O_2_), and therefore its efficacy is markedly compromised in hypoxic tumors (He et al., 2023; Sahu et al., 2020; Guo et al., 2025). To address this limitation, incorporating self‑oxygenation capabilities or oxygen modulation into PDT systems has emerged as a practical strategy to sustain reactive oxygen species (ROS) production under oxygen-limited conditions (Cheng et al., 2021; Chen et al., 2024a; Wang et al., 2025b). In this regard, metal-organic frameworks (MOFs) are well suited for multifunctional PDT, as their highly crystalline and modular architectures enable the co-integration of photosensitizing ligands with catalytically active motifs for *in situ* oxygen generation (Li et al., 2023; Zhao et al., 2022). As a compelling prototype, PCN-224(Mn) (PM), which is constructed from Zr-based clusters and Mn-porphyrin ligands (Mn-TCPP), represents a versatile scaffold for effective PDT at 660 nm (He et al., 2019). Beyond its role as a photosensitizer, the Mn-porphyrin centers function as biomimetic catalytic sites that exhibit catalase-like activity to facilitate the decomposition of endogenous H_2_O_2_ into O_2_ *in situ* (Wang et al., 2025a, Zhang and Yin, 2022). Given that elevated H_2_O_2_ is a hallmark of TME, this Mn-assisted self‑oxygenation offers a feasible route to alleviate hypoxia and support oxygen-dependent photodynamic processes, making PM attractive for overcoming hypoxia-associated PDT attenuation in HCC.

Oxygen supply alone may be insufficient, as tumor antioxidant defenses can still limit PDT efficacy. A complementary approach to potentiate PDT is to disrupt tumor redox homeostasis and thereby amplify oxidative stress (Cai et al., 2025; Sun et al., 2023). Sorafenib (Sor) is a first-line systemic drug for advanced HCC (Huang et al., 2020; Zanuso et al., 2025), yet its therapeutic benefit is often limited by intrinsic and acquired resistance (Tang et al., 2020). Sor has been reported to inhibit the cystine/glutamate antiporter, thereby restricting cystine uptake and depleting intracellular glutathione (GSH), a central antioxidant that buffers ROS (Li et al., 2020; Xue et al., 2025). Plumbagin (PLB), a bioactive naphthoquinone derived from *Plumbago zeylanica*, can further compromise cellular antioxidant defenses by downregulating glutathione peroxidase 4 (GPX4), a key enzyme that detoxifies lipid hydroperoxides and prevents ROS-driven membrane damage (Yao et al., 2023; Zhan et al., 2022; Chen et al., 2021). Because the GSH-GPX4 axis is pivotal for restraining oxidative stress, the Sor/PLB combination is expected to amplify ROS accumulation and enhance susceptibility of HCC cells to PDT-induced oxidative damage.

**Scheme 1:**
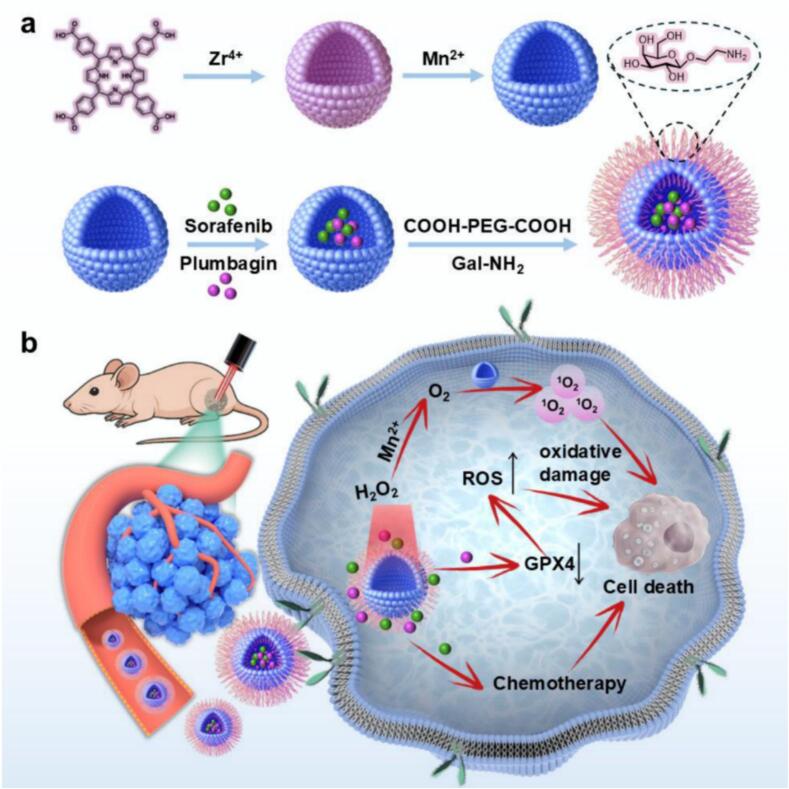
(a) Schematic illustration of the preparation of SorPLB@Gal-PM. (b) Schematic illustration of SorPLB@Gal-PM-mediated oxygen-boosted chemo–photodynamic therapy for HCC.

In this study, a galactose-modified PM-based nanoplatform (SorPLB@Gal-PM) was engineered for oxygen-boosted, synergistic chemo-photodynamic therapy against HCC (Scheme 1). In our design, PM serves not only as a photosensitizing platform for type II PDT but also as a catalytic reactor to generate O_2_ *in situ via* its intrinsic catalase-like activity, thereby alleviating hypoxia and sustaining PDT efficacy in oxygen-limited TME. The co-delivered Sor and PLB provide redox-amplifying chemotherapy. Notably, PLB-mediated GPX4 suppression weakens cellular antioxidant defenses and sensitizes HCC cells to ROS-mediated damage. Combining Sor- and PLB-mediated redox disruption with Mn-assisted oxygen modulation reinforces type II PDT efficacy in hypoxic HCC through a rational redox‑oxygen dual-regulation way. In addition, surface galactose modification enables ASGPR-mediated targeting to enhance cellular uptake and tumor accumulation (Chen et al., 2024b). Overall, this work offers a simple and effective approach for constructing Mn-porphyrinic MOF nanoplatforms with self‑oxygenation capability, and enables oxygen-boosted, ROS-amplified synergistic therapy for hypoxia-adaptive HCC treatment.

## 2 Materials and methods

### 2.1 Materials

ZrOCl_2_·4H_2_O, Tetrakis (4-carboxyphenyl) porphyrin (TCPP), Plumbagin, Sorafenib and 3,3′,5,5′-tetramethylbenzidine (TMB) were purchased from Aladdin (China). 2′,7′-dichloro fluorescein diacetate (DCFH-DA) and *N*-Hydroxy succinimide were obtained from Beyotime Biotechnology (China). HOOC-PEG2000-COOH was obtained from MeloPEG (China). The cell lines used in this study included liver cancer stem cell-like C5WN1 cells, human hepatocellular carcinoma cell lines HepG2, Huh7, and SMMC-7721, human embryonic kidney 293 (HEK293) cells, and human normal hepatocyte L02 cells.

### 2.2 Synthesis of SorPLB@Gal-PM

First, SorPLB@PM and HOOC-PEG-COOH (Mw = 2 K) (w:w = 1:1) were dispersed in water and stirred at room temperature for 4 h. The resulting mixture was then centrifuged at 12000 rpm for 30 min, and the precipitate was washed with water to obtain SorPLB@PEG-PM. SorPLB@PEG-PM, amino-modified galactose (Gal-NH_2_), EDC·HCl and NHS (w:w:w:w = 1.5:1:1.7:1) were dissolved in water and stirred at room temperature for 48 h. The mixture was then centrifuged at 12000 rpm for 30 min, and the precipitate was washed three times with water to obtain SorPLB@Gal-PM. RhB-labeled SorPLB@Gal-PM was prepared by dissolving SorPLB@Gal-PM and RhB (w:w = 2:1) in ultrapure water. The mixture was stirred for 12 h, and the product was collected after centrifugation.

### 2.3 Catalase-like activity assay

SorPLB@Gal-PM was dispersed in H_2_O_2_ solution. After different incubation times, TMB and HRP were added. Subsequently, the change in the UV–vis absorption curve of TMB was detected using a UV–Vis spectrophotometer to verify the catalase-like activity of SorPLB@Gal-PM.

### 2.4 Singlet oxygen generation capacity assay

ABDA was dissolved in PBS at a concentration of 40 μg/mL, and then SorPLB@Gal-PM was added to the ABDA solution at a concentration of 50 μg/mL. The solution was irradiated with an NIR laser, and its UV–Vis absorbance was recorded every 2 min.

### 2.5 ROS generation capacity of SorPLB@Gal-PM

2 × 10^4^ C5WN1 cells were seeded in 48-well plates. After 12 h of culture, the cells were transferred to a hypoxic environment and cultured for another 12 h. Subsequently, the cells were incubated in media containing PBS, SorPLB@Gal-PCN-224, PCN-224 (Mn) and SorPLB@Gal-PM, respectively. After 3 h, the medium was removed, and the cells were washed three times with PBS at pH 7.4. Then, medium containing 1 × 10^−5^ mol·L^−1^ DCFH-DA was added, and the cells were irradiated with 660 nm light source for 10 min. After incubating for another 15 min, the cells were washed three times with PBS at pH 7.4. Finally, the DCFH-DA fluorescence was observed using a fluorescence microscope.

### 2.6 *In vitro* targeted synergistic experiment of SorPLB@Gal-PM

C5WN1, HepG2, Huh7, and SMMC-7721 cells were seeded in 96-well plates at a density of 1 × 10^4^ cells per well. After 24 h of culture, the cells were incubated in media containing PLB, Sor, Sor + PLB, PCN-224 (Mn), SorPLB@PM, and SorPLB@Gal-PM, respectively. After 24 h, the photo-irradiation groups were irradiated with a 660 nm light (NIR) source for 10 min. After continuing to culture for 24 h, the cell viability was detected using the MTT assay. To quantify the synergistic therapeutic effect, the Bliss Independence Model was applied to analyze the interaction between targeted chemotherapy (SorPLB@Gal-PM, A) and phototherapy (PM + NIR, B). *I*_obs_ means inhibition rate of SorPLB@Gal-PM + NIR group, ΔI = *I*_A_ + *I*_B_-(*I*_A_ × *I*_B_)-*I*_exp_.

### 2.7 *In vivo* biodistribution

Four weeks old male BALB/c nude mice (18–20 g) were purchased from Slac Laboratory Animal Co. Ltd. (Hunan, China) and the protocol of all animal experiments were approved by Technology Animal Care and Use Committee of Jiangnan University (JN. No20241230b0780815[700]). For subcutaneous HCC-bearing mice model, 100 μL C5WN1 cells (2 × 10^6^) in PBS/Matrigel (v:v, 1:1) were injected subcutaneously into the lateral thigh of mice. The tumor sizes were monitored and calculated according to the formula: Tumor volume = 0.5 × (tumor width)^2^ × (tumor length). When the tumor volume reached approximately 100 mm^3^, mice were intravenously injected *via* the tail vein with 100 μL PBS containing RhB-labeled SorPLB@PM (10 mg/kg) or RhB-labeled SorPLB@Gal-PM (10 mg/kg). Fluorescence imaging was performed at 4, 8, 12, and 24 h post-injection using small living image instrument (IVIS Lumina LT Series III, PerkinElmer, USA). Major organs (heart, liver, spleen, lungs, kidneys) as well as tumor tissues were collected for *ex vivo* fluorescence imaging. All images were analyzed and normalized using Living Image software.

## 3 Results and discussions

### 3.1 Construction and characterization of SorPLB@Gal-PM

**Fig. 1:**
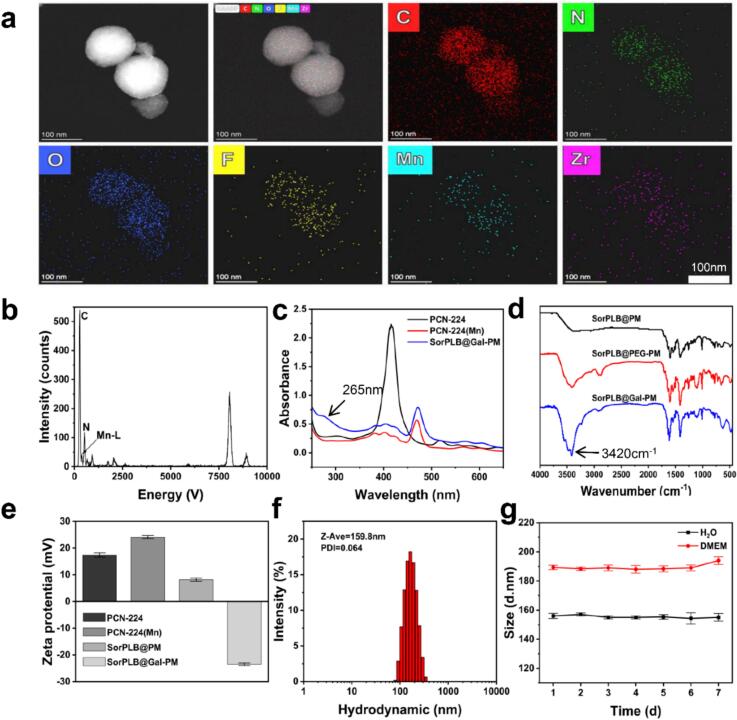
(a) TEM images of SorPLB@Gal-PM and elemental distribution of SorPLB@Gal-PM NPs mapped by HAADF-TEM spectroscopy. (b) EDS spectra of SorPLB@Gal-PM. (c) UV–vis absorption spectra of SorPLB@Gal-PM. (d) FT-IR spectra of SorPLB@Gal-PM. (e) Zeta potential of PCN-224, PM, SorPLB@PM and SorPLB@Gal-PM, mean ± SD (*n* = 3). (f) Hydrodynamic size distribution of SorPLB@Gal-PM. (g) Long-term hydrodynamic size stability of SorPLB@Gal-PM, mean ± SD (n = 3).

PM was synthesized from ZrOCl_2_·H_2_O, TCPP, and benzoic acid in DMF following a reported procedure (He et al., 2019). Sor and PLB were then loaded into the framework to obtain SorPLB@PM. HOOC-PEG-COOH was subsequently grafted onto the nanoparticles *via* electrostatic adsorption, providing exposed carboxyl termini for further modification. Amine-modified galactose (1-O-(2-aminoethyl)-β-d-galactopyranose, Gal-NH_2_) was synthesized according to a previous report (Hu et al., 2020). The carboxyl-functionalized SorPLB@PM was then conjugated with Gal through EDC-mediated coupling to yield SorPLB@Gal-PM. High-Angle Annular Dark-Field Scanning Transmission Electron Microscopy (HAADF-TEM) image showed a uniform spherical morphology (Fig. 1a). Energy Dispersive X-ray Spectroscopy Mapping (EDS) results confirmed the homogeneous distribution of Mn and the presence of fluorine, indicating successful Mn^2+^ coordination and Sor loading (Fig. 1b).

The successful synthesis of PM and the loading of Sor and PLB were confirmed by UV–vis spectroscopy (Fig. 1c). Compared with PCN-224, PM exhibited a noticeable shift from 416 nm to 472 nm, which was attributed to the coordination of Mn^2+^ within the TCPP framework. In addition, SorPLB@Gal-PM displayed a characteristic absorption band at 265 nm, supporting successful drug incorporation. Sor@Gal-PM and PLB@Gal-PM were also prepared separately, and both showed a characteristic absorption peak at 265 nm (Fig. S1a, b). Fourier Transform infrared spectroscopy (FTIR) spectrum displayed a distinct band at 3420 cm^−1^ due to the O—H stretching vibration of Gal (Fig. 1d), confirming the successful conjugation of the Gal ligand.

The zeta potential decreased from +24.1 mV (PM) to +8.2 mV (SorPLB@PM) after drug loading, and further reversed to −23.5 ± 0.45 mV after Gal functionalization (SorPLB@Gal-PM) (Fig. 1e), indicating successful surface modification. Dynamic light scattering (DLS) showed that SorPLB@Gal-PM had an average hydrodynamic diameter of 159.2 ± 1.5 nm with a PDI of 0.064 (Fig. 1f), suggesting a narrow size distribution and good uniformity. Moreover, SorPLB@Gal-PM was dispersed in water and DMEM, and the hydrodynamic size was monitored by DLS over 7 days. No obvious change in particle size was observed throughout the measurement period, indicating good stability (Fig. 1g).

### 3.2 Drug release and photodynamic properties

**Fig. 2:**
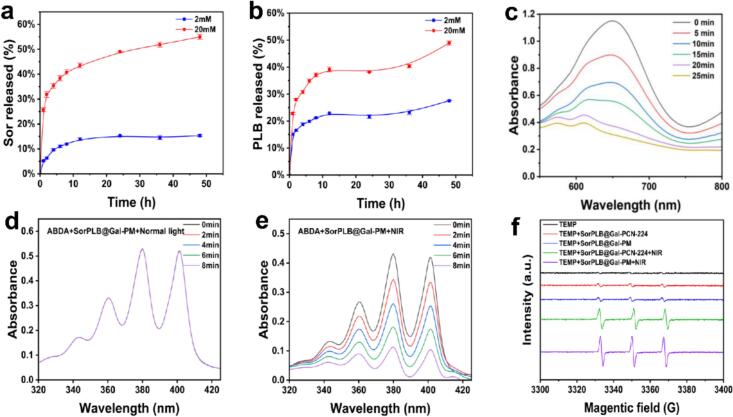
(a, b) Cumulative release profiles of Sor (a) and PLB (b) from SorPLB@Gal-PM in PBS containing 2 mM or 20 mM phosphate (mean ± SD, *n* = 3). (c) Time-dependent UV–vis spectra of the TMB/HRP system in the presence of SorPLB@Gal-PM and H_2_O_2_. (d, e) UV–vis spectra of ABDA in the presence of SorPLB@Gal-PM under (d) normal light or (e) NIR irradiation. (f) ESR spectra of SorPLB@Gal-PM under different treatments using TEMP as the spin-trapping agent.

Sor and PLB were co-loaded into PM by mixing PM with Sor and PLB at a series of feed ratios. Standard calibration curves for Sor and PLB were established (Fig. S1c, d). The drug loading content (DLC) and drug encapsulation efficiency (DEE) were quantified by HPLC. At a feed ratio of PM: Sor: PLB = 1:2:1, the DLC and DEE of Sor were 12.41% ± 0.67% and 9.57% ± 0.83%, respectively, while those of PLB were 7.51% ± 0.78% and 13.06% ± 0.91%, respectively. The co-loaded mass ratio of Sor to PLB in SorPLB@Gal-PM was approximately 1.65:1 based on HPLC quantification. Since intracellular phosphate levels are generally higher than those in the extracellular milieu (He et al., 2014), the phosphate-triggered release behavior of SorPLB@Gal-PM was investigated in PBS containing 2 mM or 20 mM phosphate. SorPLB@Gal-PM exhibited a markedly accelerated release under the high-phosphate condition (Fig. 2a, b). After 48 h, the cumulative release of Sor and PLB reached 54.98% ± 1.04% and 48.91% ± 0.78% in 20 mM phosphate PBS, respectively. In contrast, only 15.37% ± 0.37% (Sor) and 27.53% ± 0.17% (PLB) were released in 2 mM phosphate PBS over the same period. These results demonstrate a phosphate-responsive release profile, suggesting that SorPLB@Gal-PM may facilitate preferential drug release in phosphate-rich intracellular environments after cellular uptake, thereby reducing premature leakage during circulation.

To evaluate the Mn-mediated catalase-like activity of SorPLB@Gal-PM, an HRP/TMB assay was performed (Yu et al., 2023). The results showed that H_2_O_2_ oxidizes TMB under HRP catalysis to generate oxTMB with a characteristic absorption at 652 nm. Therefore, attenuation of the 652 nm signal reflects a decreased effective H_2_O_2_ level in the system. The TMB-associated absorbance at 652 nm gradually decreased after adding SorPLB@Gal-PM (Fig. 2c), indicating continuous H_2_O_2_ consumption. The endpoints of different treatments showed that SorPLB@Gal-PM produced a markedly lower signal than the H_2_O_2_ control (Fig. S2a). Notably, SorPLB@Gal-PM exhibited stronger H_2_O_2_-scavenging effect than PM under the same conditions, suggesting that drug loading and surface modification preserved or even enhanced the catalytic performance. The PM-based and related Mn-porphyrinic MOF systems have been previously reported to catalyze the decomposition of H_2_O_2_ into O_2_ (Feng et al., 2022; Xu et al., 2021). A visible bubble formation was observed upon introducing SorPLB@Gal-PM into the H_2_O_2_ solution (Fig. S2b), which thus supports O_2_ generation of SorPLB@Gal-PM. Collectively, these results support the catalase-mimicking activity primarily originating from the PM framework.

The photodynamic ROS generation capability of SorPLB@Gal-PM was further assessed using ABDA as a singlet oxygen (^1^O_2_) probe. In the presence of SorPLB@Gal-PM, NIR irradiation induced a pronounced decrease in ABDA absorbance (Fig. 2d, e and Fig. S2c, d), suggesting ^1^O_2_ generation upon NIR activation. ESR spectroscopy using 2,2,6,6-tetramethylpiperidine (TEMP) as the spin-trapping agent showed the characteristic 1:1:1 triplet signal after NIR irradiation (Fig. 2f), providing additional evidence for ^1^O_2_ production by SorPLB@Gal-PM. Together with its catalase-like O_2_-evolving activity, SorPLB@Gal-PM could locally replenish oxygen and alleviate hypoxia, thereby mitigating hypoxia-associated limitations and enhancing ^1^O_2_-mediated PDT under hypoxic conditions.

### 3.3 *In vitro* targeting ability and cytotoxicity of SorPLB@Gal-PM

**Fig. 3:**
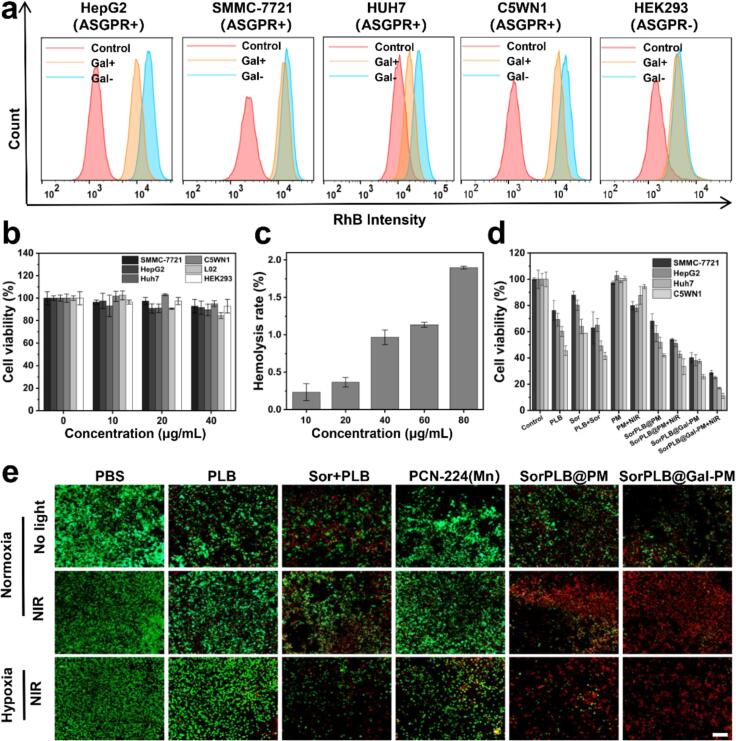
(a) Flow cytometry histograms of RhB fluorescence in cells treated with RhB-labeled SorPLB@Gal-PM with (Gal^+^) or without (Gal^−^) galactose (1 mM) competition; untreated cells served as control. (b) Cell viability of HepG2, C5WN1, Huh7, SMMC-7721, L02 and HEK293 cells incubated with Gal-PM at various concentrations for 48 h. Mean ± SD (*n* = 3). (c) Hemolysis of red blood cells induced by SorPLB@Gal-PM. Mean ± SD (n = 3). (d) Cell viability of SMMC-7721, HepG2, Huh7 and C5WN1 cells after different treatments for 48 h. Mean ± SD (n = 3). (e) Calcein-AM/propidium iodide (PI) double staining of SMMC-7721 cells after different treatments. Scale bar: 200 μm. (For interpretation of the references to colour in this figure legend, the reader is referred to the web version of this article.)

The ASGPR-mediated uptake of SorPLB@Gal-PM was evaluated in ASGPR-positive HCC cells (HepG2, SMMC-7721, Huh7, and C5WN1) and ASGPR-negative HEK293 cells by flow cytometry and fluorescence microscopy. Cells were pretreated with free galactose (1 mM, Gal^+^) to competitively block ASGPR, while untreated cells served as the non-competition group (Gal^−^) (Zhao et al., 2017; Zhang et al., 2023b). In HCC cells, the Gal- group showed a clear right shift in fluorescence histograms compared with the Gal+ group (Fig. 3a), whereas no obvious difference was observed in HEK293 cells. Fluorescence imaging showed the same trend (Fig. S3). These results confirmed that SorPLB@Gal-PM enhanced uptake in HCC cells primarily through Gal–ASGPR recognition and receptor-mediated endocytosis.

To assess biocompatibility, the blank nanocarrier Gal-PM was incubated with Huh7, C5WN1, SMMC-7721, HepG2, HEK293, L02 cells for 48 h (Fig. 3b). Cell viabilities remained above 80% even at 40 μg/mL, indicating good cytocompatibility of the carrier. Hemocompatibility was further examined by a hemolysis assay (Fig. 3c), and the hemolysis ratio remained below 2% even at 80 μg/mL of SorPLB@Gal-PM.

The *in vitro* anti-cancer efficacy of SorPLB@Gal-PM was evaluated by 3-(4,5-dimethylthiazol-2-yl)-2,5-diphenyltetrazolium bromide (MTT) assay in HepG2, Huh7, C5WN1, and SMMC-7721 cells (Fig. 3d and Fig. S4b). Treatment groups included: PBS (vehicle control), PLB (free PLB), Sor (free Sor), Sor + PLB (free drug combination), PM (blank carrier), SorPLB@PM (Sor/PLB-loaded PM), SorPLB@Gal-PM (galactose-modified SorPLB@PM), SorPLB@PM + NIR (SorPLB@PM with irradiation), and SorPLB@Gal-PM + NIR (SorPLB@Gal-PM with irradiation). SorPLB@Gal-PM induced pronounced, dose-dependent cytotoxicity in all tested HCC cell lines (Fig. S4a). In contrast, normal cells L02 and HEK293 exhibited comparatively higher viability under the same treatments (Fig. S4b), indicating improved *in vitro* selectivity and reduced cytotoxicity. Under the same drug-equivalent conditions, SorPLB@Gal-PM exhibited stronger cell-killing effects than free drugs and SorPLB@PM (Fig. 3d), indicating enhanced efficacy after nano-formulation and Gal-mediated targeting. Importantly, SorPLB@Gal-PM + NIR produced the lowest cell viability among all groups. Synergy was further quantified using the Bliss independence model (Meyer et al., 2020). Calculated from the inhibition rates of SorPLB@Gal-PM (chemotherapy), PM + NIR (phototherapy), and SorPLB@Gal-PM + NIR (combined treatment), the chemo-phototherapy combination showed positive synergy scores (ΔI) across all tested HCC cell lines (Table S1), with ΔI values of +3.25% (SMMC-7721), +4.71% (HepG2), +15.87% (Huh7), and + 13.28% (C5WN1). These *in vitro* results support SorPLB@Gal-PM as a promising nanoplatform for HCC-targeted delivery and synergistic chemo-phototherapy. Calcein-AM/PI staining was also performed (Fig. 3e) (Zhang et al., 2021). SorPLB@Gal-PM + NIR showing the most pronounced shift toward red fluorescence, indicating extensive cell death. Notably, under hypoxic conditions, both SorPLB@Gal-PM and SorPLB@PM still induced substantial cell killing upon NIR irradiation. This retained efficacy may be associated with the Mn-containing PM framework, which exhibits catalase-like activity to decompose endogenous H_2_O_2_ and generate O_2_ *in situ*, thereby partially improving local oxygen availability. Collectively, these results suggest that SorPLB@Gal-PM enables potent chemo-phototherapy under different oxygen conditions.

### 3.4 Intracellular hypoxia alleviation and PDT activity of SorPLB@Gal-PM

**Fig. 4:**
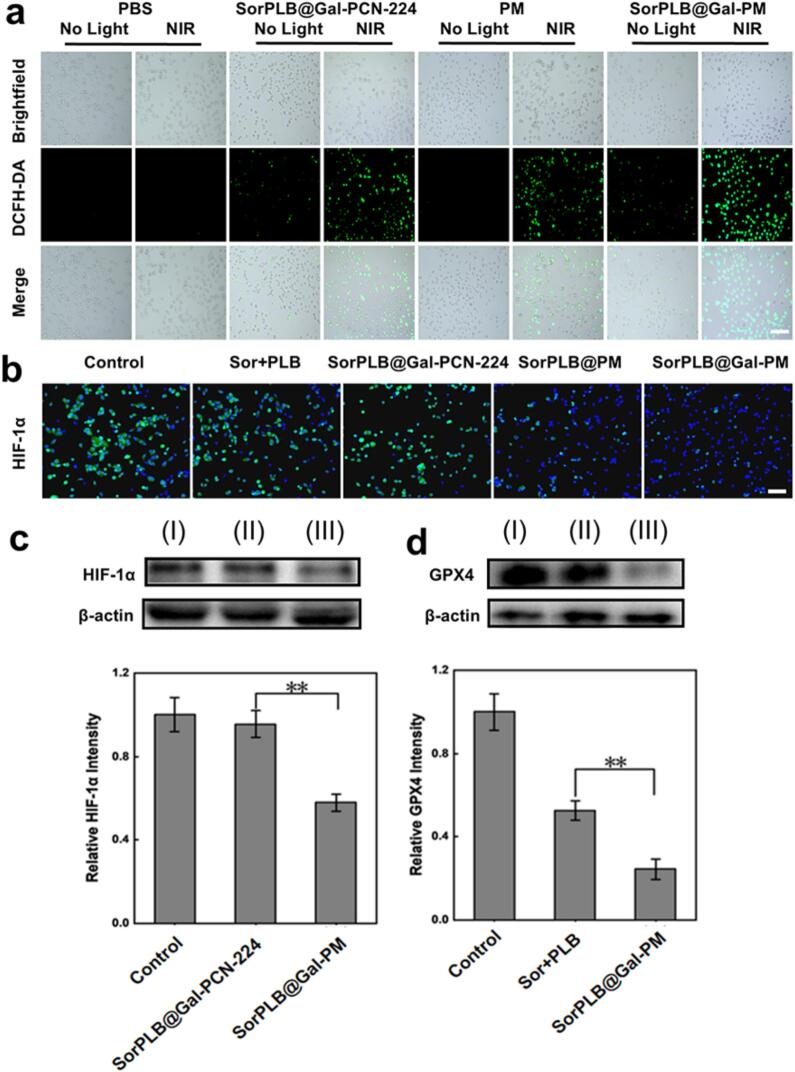
(a) Images of intracellular ROS levels in C5WN1 cells after different treatments. Scale bar: 100 μm. (b) Immunofluorescence images of HIF-1α in C5WN1 cells after different treatments. Scale bar: 100 μm. (c) Western blot analysis of HIF-1α in C5WN1 cells and corresponding densitometric quantification. (I) Control, (II) SorPLB@Gal-PCN-224, (III) SorPLB@Gal-PM. (d) Western Blot analysis of GPX4 in C5WN1 cells and corresponding densitometric quantification. (I) Control, (II) Sor + PLB, (III) SorPLB@Gal-PM. Data are presented as mean ± SD (*n* = 3). *p < 0.05, **p < 0.01.

To verify ROS generation and the photodynamic performance of SorPLB@Gal-PM under hypoxic conditions, C5WN1 cells were pretreated under hypoxia and then incubated with PBS, SorPLB@Gal-PCN-224, PM, or SorPLB@Gal-PM, followed with/without irradiation. Intracellular ROS levels were assessed using the DCFH-DA probe. Negligible fluorescence was observed in the PBS (± NIR) groups, indicating that NIR irradiation alone did not induce detectable ROS (Fig. 4a). In contrast, obvious ROS signals appeared in the PM + NIR and SorPLB@Gal-PCN-224 + NIR groups, while the SorPLB@Gal-PM + NIR group exhibited the strongest green fluorescence, indicating markedly enhanced ROS generation of constructed nanoplatform upon irradiation. Quantitative analysis further showed that the ROS level in the SorPLB@Gal-PM + NIR group was significantly higher than that in SorPLB@Gal-PCN-224 + NIR and PM + NIR groups by approximately 3.4-fold and 2.9-fold, respectively (Fig. S5). Collectively, these results demonstrate that the Mn-containing framework supports ROS generation under hypoxia upon NIR activation, while Sor/PLB chemotherapy further weakens antioxidant defenses, together resulting in a pronounced oxidative burst.

HIF-1α is a hypoxia-responsive transcription factor that is stabilized under low-oxygen conditions and is commonly used as an indicator of cellular hypoxia (Mendez-Blanco et al., 2018; Kim et al., 2017). To evaluate whether SorPLB@Gal-PM could improve the hypoxic status of tumor cells, hypoxia-pretreated C5WN1 cells were treated with SorPLB@Gal-PCN-224 (non-Mn control) or SorPLB@Gal-PM, and HIF-1α levels were analyzed by immunofluorescence and western blot. SorPLB@Gal-PM markedly reduced HIF-1α fluorescence compared with the control and SorPLB@Gal-PCN-224 groups (Fig. 4b). Consistently, HIF-1α expression in the SorPLB@Gal-PM group decreased to 57.89 ± 4.17% of the control (Fig. 4c), whereas SorPLB@Gal-PCN-224 exhibited only a minimal reduction (95.55 ± 6.42% of the control). These results suggest that the Mn-containing SorPLB@Gal-PM is effective in alleviating hypoxia at the cellular level, which is consistent with the catalase-like activity of Mn that can decompose endogenous H_2_O_2_ to generate O_2_ *in situ*.

In addition, GPX4 is a key antioxidant enzyme that suppresses lipid peroxidation and limits ROS-driven oxidative damage (Liu et al., 2024). Western blot analysis showed that SorPLB@Gal-PM induced a significantly stronger downregulation of GPX4 expression level than the free-drug combination by western blot (Fig. 4d). Specifically, GPX4 levels decreased to 24.38 ± 4.94% of the control in the SorPLB@Gal-PM group, compared with 52.52 ± 4.57% in the Sor + PLB group. This enhanced GPX4 suppression implies that nano-formulation facilitates the intracellular action of Sor + PLB, thereby weakening antioxidant defenses, amplifying oxidative stress, and contributing to improved tumor cell killing.

### 3.5 *In vivo* biodistribution of SorPLB@Gal-PM

**Fig. 5:**
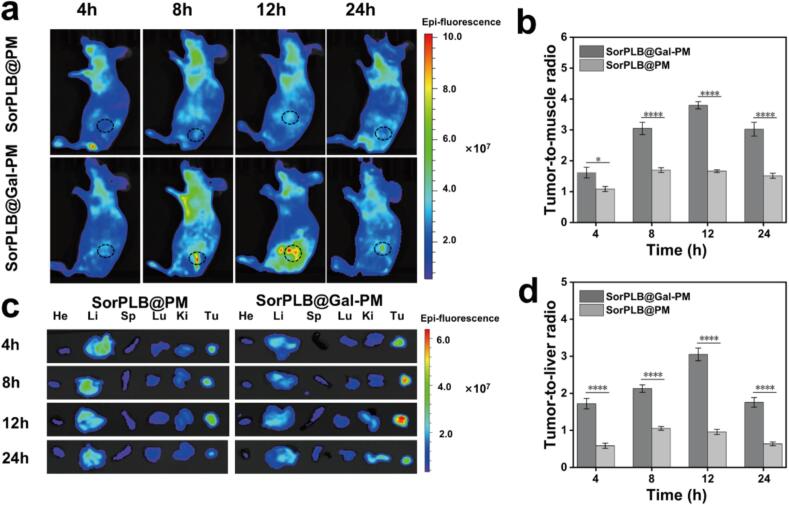
(a) Images of RhB-labeled SorPLB@PM or RhB-labeled SorPLB@Gal-PM at 4, 8, 12, and 24 h after intravenous injection (10 mg/kg). The dashed circles indicate the tumor regions. (b) Tumor-to-muscle ratio (T/M) of SorPLB@Gal-PM and SorPLB@PM on C5WN1 tumor-bearing mice at, 4, 8, 12, and 24 h post-injection. (c) *Ex vivo* images of harvested major organs and tumors at 4, 8, 12, and 24 h post-injection (He, heart; Li, liver; Sp, spleen; Lu, lung; Ki, kidney; Tu, tumor). (d) Tumor-to-liver ratio (T/L) of SorPLB@Gal-PM and SorPLB@PM on C5WN1 tumor-bearing mice at, 4, 8, 12, and 24 h post-injection.

The tumor-targeting capacity and biodistribution of SorPLB@Gal-PM and SorPLB@PM were evaluated in C5WN1 tumor-bearing nude mice using RhB labeling for *in vivo* tracking (Hu et al., 2022). When the tumor volume reached 100 mm^3^, mice were intravenously injected with RhB-labeled SorPLB@Gal-PM or SorPLB@PM, and fluorescence images were acquired at 4, 8, 12, and 24 h post-injection (Fig. 5a). Both formulations displayed detectable fluorescence in the tumor region, whereas SorPLB@Gal-PM showed stronger and more persistent tumor-associated signals, reaching a maximum at 12 h and remaining clearly observable at 24 h. Specifically, the tumor-to-muscle (T/M) ratio of SorPLB@Gal-PM peaked at 12 h (approximately 3.8), which was 2.2-fold higher than that of SorPLB@PM (approximately 1.7) (Fig. 5b). These results suggest enhanced tumor accumulation and retention after galactose modification.

*Ex vivo* imaging of excised organs and tumors further confirmed these observations (Fig. 5c). Clear fluorescence signals were detected in tumor tissues after SorPLB@Gal-PM administration at all examined time points. Notably, the tumor-to-liver ratio (T/L) of SorPLB@Gal-PM was significantly higher than that of SorPLB@PM at all examined time points (*e.g.*, 3.1 *vs.* 0.9 at 12 h), indicating improved tumor selectivity with galactose modification (Fig. 5d). SorPLB@Gal-PM exhibited enhanced tumor fluorescence and improved tumor retention, whereas SorPLB@PM showed more pronounced liver-associated signals relative to the tumor. Collectively, these results indicate that galactose functionalization promotes tumor accumulation and retention of SorPLB@Gal-PM *in vivo*.

### 3.6 *In vivo* anti-HCC therapeutic efficacy of SorPLB@Gal-PM

**Fig. 6:**
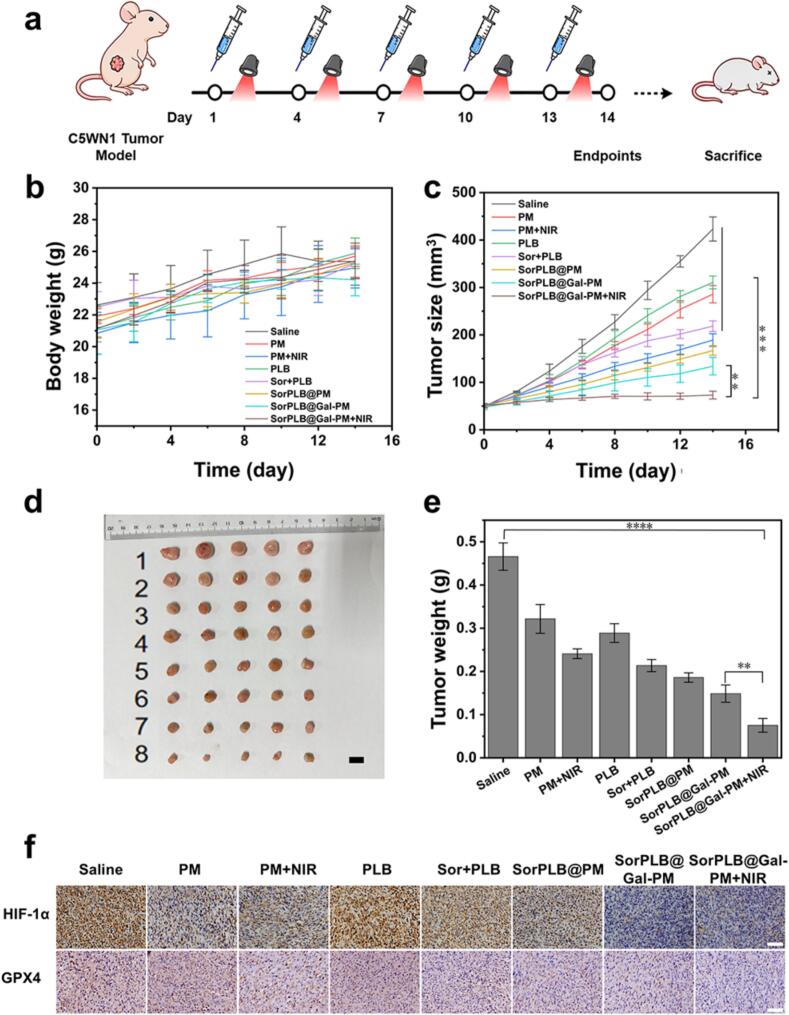
(a) Schematic illustration of the *in vivo* treatment schedule in C5WN1 tumor-bearing nude mice. (b) Weight changes of mice body during treatment. Means ± SD, *n* = 5. (c) Tumor growth curves of different treatment groups. (d) Representative images of excised tumors on day 14. Scale bar: 1 cm. (1) Saline, (2) PM, (3) PM + NIR, (4) PLB, (5) Sor + PLB, (6) SorPLB@PM, (7) SorPLB@Gal-PM and (8) SorPLB@Gal-PM + NIR. (e) Tumor weights on day 14. (e) IHC staining of HIF-1α and GPX4 in tumor sections after 14 days treatment. (scale bar: 100 μm). Data are presented as mean ± SD (n = 5). *p < 0.05, **p < 0.01, ***p < 0.001.

The *in vivo* anti-HCC therapeutic efficacy of SorPLB@Gal-PM was evaluated in C5WN1 tumor-bearing BALB/c nude mice (Fig. 6a). When tumors reached 50 mm^3^, mice were randomly assigned into eight groups (*n* = 5): Saline (vehicle control), PLB (free drug), Sor + PLB (free drug combination), PM (blank carrier), PM + NIR (PM with irradiation), SorPLB@PM (Sor/PLB-loaded PM), SorPLB@Gal-PM (galactose-modified SorPLB@PM), and SorPLB@Gal-PM + NIR (SorPLB@Gal-PM with irradiation). No obvious body-weight loss was observed in any group throughout the treatment period (Fig. 6b), indicating good tolerability. Tumor growth was monitored during treatment (Fig. 6c). At the endpoint (day 14), tumors were excised for representative photographs and weight measurements (Fig. 6d and Fig. 6e). SorPLB@Gal-PM + NIR exhibited the most potent tumor suppression, yielding a tumor inhibition rate (TIR) of 83.70 ± 4.12%, which was much higher than that of SorPLB@Gal-PM (68.33 ± 4.87%). The Sor + PLB group and SorPLB@PM group only achieved TIRs of 55.29 ± 3.49% and 60.58 ± 2.64%, respectively. Consistently, the excised tumor photographs further confirmed the smallest tumor sizes in the SorPLB@Gal-PM + NIR group. H&E staining of tumor tissues further corroborated these findings, showing the most significant area of tumor cell necrosis and structural damage in SorPLB@Gal-PM + NIR compared to other treatment groups (Fig. S6). Collectively, these results demonstrate that SorPLB@Gal-PM achieved superior antitumor efficacy with favorable tolerability *in vivo*.

Immunohistochemical analysis was conducted to evaluate treatment-related changes in the protein expression patterns of HIF-1α and GPX4 within tumors. Compared with the saline control, tumors treated with SorPLB@Gal-PM (especially with NIR) exhibited markedly reduced HIF-1α and GPX4 staining (Fig. 6f). This decline is consistent with the alleviated hypoxia stress provided by the intrinsic catalase-like activity of the PM framework, which effectively weakens the tumor's antioxidant defense and sensitizes cells to the combined therapy.

Finally, the *in vivo* biosafety of the system was thoroughly evaluated. H&E staining of major organs showed no noticeable pathological abnormalities across all treatment groups (Fig. S7). Furthermore, blood biochemistry and hematology parameters remained within normal physiological ranges (Fig. S8), collectively supporting the favorable biosafety and low systemic toxicity of SorPLB@Gal-PM.

## 4 Conclusion

In conclusion, a self-oxygenating Mn-porphyrinic MOF nanoplatform (SorPLB@Gal-PM) was constructed based on the PM framework for oxygen-boosted chemo–photodynamic therapy of HCC. The Mn-centered catalase-like activity enabled *in situ* O_2_ generation from endogenous H_2_O_2_, thereby alleviating hypoxia and helping sustain oxygen-dependent type II PDT under irradiation. Meanwhile, Sor/PLB co-delivery together with GPX4 suppression weakened antioxidant defenses and promoted oxidative stress amplification. Therefore, SorPLB@Gal-PM markedly enhanced ROS generation in hypoxic tumor cells upon irradiation, downregulated HIF-1α and GPX4, and translated these molecular effects into pronounced tumor growth inhibition *in vivo* while maintaining favorable biosafety profiles. Overall, this work provided a feasible approach that integrated Mn-assisted self-oxygenation and ROS-amplified chemo-PDT for improved treatment of hypoxia-challenged HCC.

## CRediT authorship contribution statement

**Xiang Wang:** Writing – original draft, Visualization, Investigation. **Hengrui Li:** Writing – original draft, Validation, Investigation. **Le Wang:** Investigation. **Yihan Ma:** Investigation, Data curation. **Miao Qin:** Investigation. **Ruonan Sun:** Validation. **Jing Hu:** Writing – review & editing, Supervision, Methodology, Funding acquisition, Conceptualization.

## Declaration of competing interest

The authors declare that they have no known competing financial interests or personal relationships that could have appeared to influence the work reported in this paper.

## Data Availability

Data will be made available on request.
