# Supplementary material for: Self-oxygenating PCN-224(Mn) co-delivers sorafenib and plumbagin to boost chemo-photodynamic therapy in hepatocellular carcinoma
Source: Int J Pharm X. 2026 Apr 28;11:100554. doi: 10.1016/j.ijpx.2026.100554 (PMC13185938; doi:10.1016/j.ijpx.2026.100554)
Supplement: Supplementary file 1 — Supplementary material [file mmc1.docx]

**Supporting Information**

**Self-Oxygenating PCN-224(Mn) Co-Delivers Sorafenib and Plumbagin to Boost Chemo-Photodynamic Therapy in Hepatocellular Carcinoma**

**Xiang Wang^a,c,#^, Hengrui Li^a,#^, Le Wang^b^, Yihan Ma^a^, Miao Qin^a^, Ruonan Sun^a^, Jing Hu^a,d*^**

^a^Wuxi School of Medicine, Jiangnan University, Wuxi 214122, China.

^b^School of Biotechnology and Key Laboratory of Carbohydrate Chemistry and Biotechnology of Ministry of Education, Jiangnan University, Wuxi 214122, China.

^c^School of Food Science and Technology, Jiangnan University, Wuxi 214122, China.

^d^Institute of Future Food Technology, JITRI, No.19 Wenzhuang Road, Yixing 214200, China.

*Corresponding authors.

E-mail addresses: hujing@jiangnan.edu.cn (Jing Hu).

^#^These authors contributed equally to this work.

**Methods**

**Synthesis of PCN-224**

ZrOCl_2_·8H_2_O, TCPP, and benzoic acid (w:w:w=3:1:28) were dissolved in 50 mL DMF. The mixture was stirred at 90 ℃ under dark conditions for 5 h. After the reaction was complete, the precipitate was collected by centrifugation at 12000 rpm and 4 ℃for 30 min. The precipitate was then washed three times sequentially with DMF and water using the same centrifugation conditions to obtain PCN-224. The PCN-224 was stored in a 4 ℃ refrigerator under dark conditions.

**Synthesis of PCN-224(Mn) (PM)**

PCN-224 and MnCl_2_·4H_2_O (w:w=2:1) were dispersed in DMF. The mixture was stirred at 120 ℃ under dark conditions for 3 h. After the reaction was complete, the precipitate was collected by centrifugation at 12000 rpm and 4 ℃ for 30 min. The precipitate was then washed three times sequentially with DMF and water using the same centrifugation conditions to obtain PM. PM was stored in a 4 ℃ refrigerator under dark conditions.

**Synthesis of SorPLB@PM**

First, PM, Sor, and PLB (w:w:w=1:2:1) were dispersed in methanol and stirred at room temperature under dark conditions for 12 h. The resulting mixture was centrifuged at 12000 rpm for 30 min, and the precipitate was washed three times with water to obtain SorPLB@PM.

**Drug loading capacity assay**

During the synthesis of SorPLB@Gal-PM, the collected supernatant after centrifugation was analyzed by HPLC to determine the content of Sor and PLB, which was used to calculate the encapsulation efficiency of SorPLB@Gal-PM.

After the synthesis of SorPLB@Gal-PM, the drugs were acid-digested with hydrochloric acid, and the resulting solution was analyzed by HPLC to determine the content of Sor and PLB, which was used to calculate the drug loading capacity of SorPLB@Gal-PM.

DLC (%) = (Amount of drug loaded into the nanomaterial / Total mass of the drug-loaded nanomaterial) ×100%

DEE (%) = (Amount of drug loaded into the nanomaterial / Total mass of the drug input)×100%

**Drug release assay**

Given that the intracellular phosphate concentration in tumor cells is much higher than the extracellular phosphate concentration, SorPLB@Gal-PM was dispersed in PBS with phosphate concentrations of 2 mM and 20 mM, and then shaken in a constant temperature shaker at 37 ℃. The supernatant was collected at 1, 2, 4, 6, 8, 12, 24, 36, and 48 h. After centrifugation, the content of Sor and PLB was measured by HPLC to calculate the drug release rate of Sor and PLB.

**Stability assay**

SorPLB@Gal-PM was dispersed in deionized water and DMEM containing 10% FBS, respectively, and its hydrodynamic particle size was continuously measured for seven days.

**Cell culture method**

C5WN1, HepG2，Huh7, and HEK293 cells were cultured in high-glucose DMEM medium (containing 10% heat-inactivated serum, 1% penicillin, and 1% streptomycin). L02 and SMMC-7721 cells were cultured in RPMI 1640 medium (containing 10% heat-inactivated serum, 1% penicillin, and 1% streptomycin). All cells were cultured in an incubator at 37 ℃ with 5% CO_2_.

**Blank material toxicity analysis**

C5WN1, HepG2, Huh7, HEK293, L02, and SMMC-7721 cells were seeded in 96-well plates at a density of 1×10^4^ cells per well. After 24 h of culture, the medium was removed, and drug-free Gal-PM at concentrations of 0, 10, 20, 40 μg·mL^−1^ was co-incubated with all the above cells for 48 h. Subsequently, the cell viability was detected using the MTT assay.

**Cell cytotoxicity analysis of SorPLB@Gal-PM**

C5WN1, HepG2, Huh7, HEK293, L02, and SMMC-7721 cells were seeded in 96-well plates at a density of 1×10^4^ cells per well. After 24 h of culture, SorPLB@Gal-PM at concentrations of 0, 5, 10, 20, 40 μg·mL^−1^ was incubated with all the above cells for 48 h. Subsequently, the cell viability was detected using the MTT assay.

Calcein-AM/PI live/dead staining was also used to detect cell viability. SMMC-7721 cells were seeded in 96-well plates at a density of 1×10^4^ cells per well. After 24 h of culture, the cells were incubated in media containing PBS, PLB, Sor, Sor + PLB, PM, SorPLB@PM, and SorPLB@Gal-PM, respectively. After 24 h, the photo-irradiation groups were irradiated with a NIR for 10 min. After continuing to culture for 24 h, the cells were co-stained with Calcein-AM/PI for 30 min. Finally, the cells were rinsed once with PBS and observed under a fluorescence inverted microscope to assess cell viability.

**Targeted Delivery Experiment of SorPLB@Gal-PM**

8×10^4^ HepG2, Huh7, SMMC-7721, C5WN1, and HEK293 cells were seeded in 6-well plates. After 12 h of culture, 1×10^-3^ mol·L^−1^ galactose was added to the galactose competition group, and culture was continued for another 12 h. The cells were then washed three times with PBS solution at pH 7.4. Subsequently, the cells were incubated in medium containing 20 μg·mL^−1^ SorPLB@Gal-PM for 3h. After removing the medium, the cells were digested with trypsin, centrifuged, and resuspended in PBS solution at pH 7.4. This process was repeated three times to thoroughly wash off extracellular trypsin and nanomaterials, eliminating their interference with fluorescence. Finally, the cells were resuspended in PBS solution at pH 7.4, transferred to flow tubes, and the fluorescence intensity of each group of cells was detected using a flow cytometer.

***In vivo* anti-HCC capacity of SorPLB@Gal-PM**

When the tumor volume reached 50 mm^3^, mice were randomly divided into the Saline, PLB, Sor + PLB, PM, PM + NIR, SorPLB@PM, SorPLB@Gal-PM and SorPLB@Gal-PM + NIR (n=5). 100 μL of each test solution were injected via the tail vein on days 1, 4, 7, 10 and 13. The NIR groups received NIR laser irradiation for 10 min after 24 h. The body weight and tumor volume of the mice were recorded during the treatment period. On day 14, the mice were anesthetized with isoflurane and euthanized with carbon dioxide. The tumors and major organs (heart, liver, spleen, lungs, and kidneys) were dissected. Tumors were photographed and weighed. Hematoxylin and eosin (H&E) staining was used to analyze the changes in cell morphology in different organ tissues and tumors. Blood indexes and liver/kidney function indexes of each group of mice were analyzed using an automatic biochemical analyzer and an automatic blood cell analyzer to evaluate the safety and toxicity of SorPLB@Gal-PM in tumor-bearing mice.

**Statistical analysis**

Data in this study were presented as the means with standard errors (means ± SD). Differences among groups were evaluated using unpaired Student's t-test and one-way analysis of variance (ANOVA) with GraphPad Prism 7.0 software. The P < 0.05 was considered statistically significant.

**Figures and Tables**


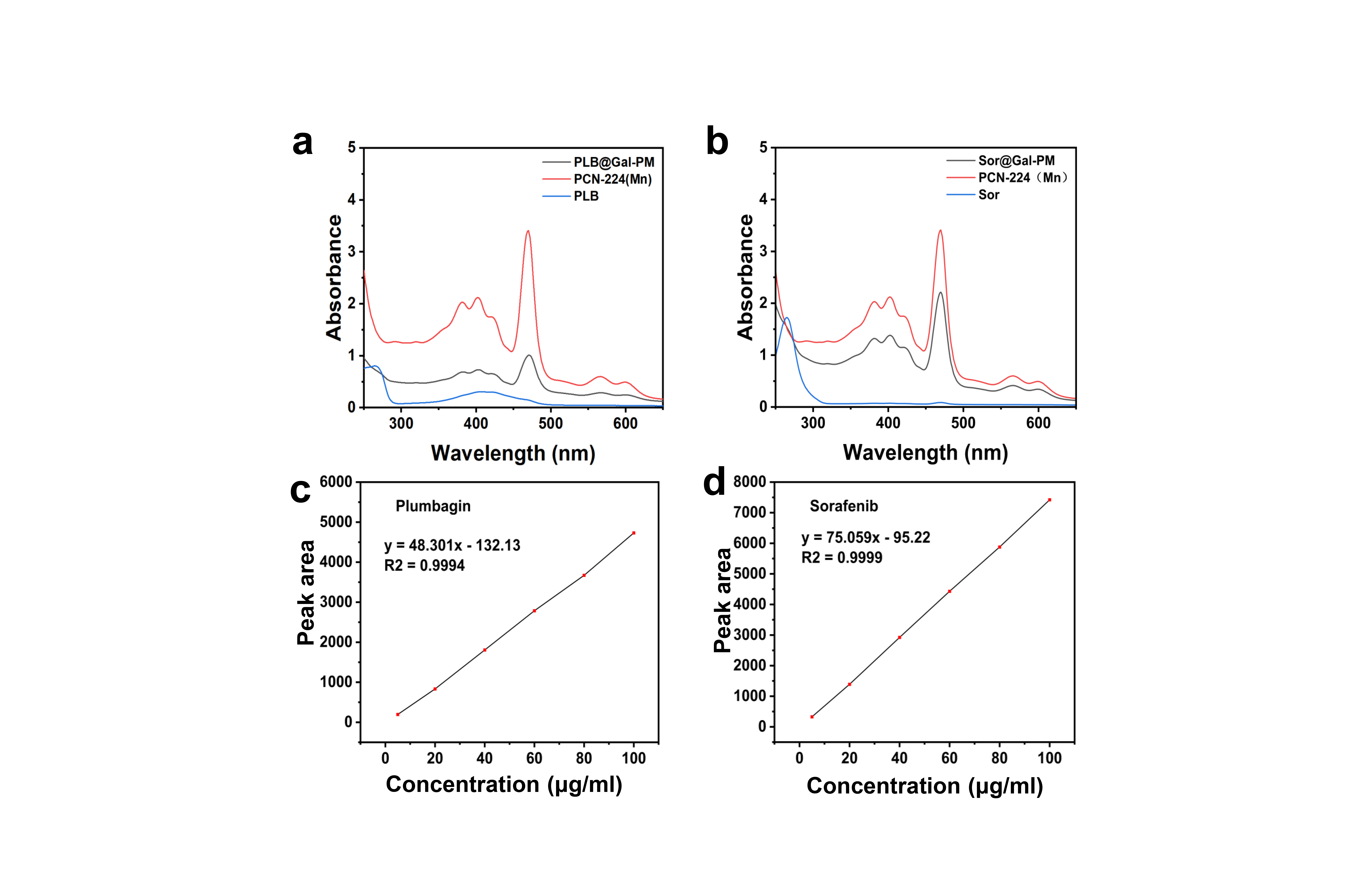


**Fig S1**. (a) UV-vis absorption spectra of PLB@Gal-PM. (b) UV-vis absorption spectra of Sor@Gal-PM.(c) The standard curve of PLB in MeOH at the wavelength of 265 nm. (d) The standard curve of Sor in MeOH at the wavelength of 265 nm.


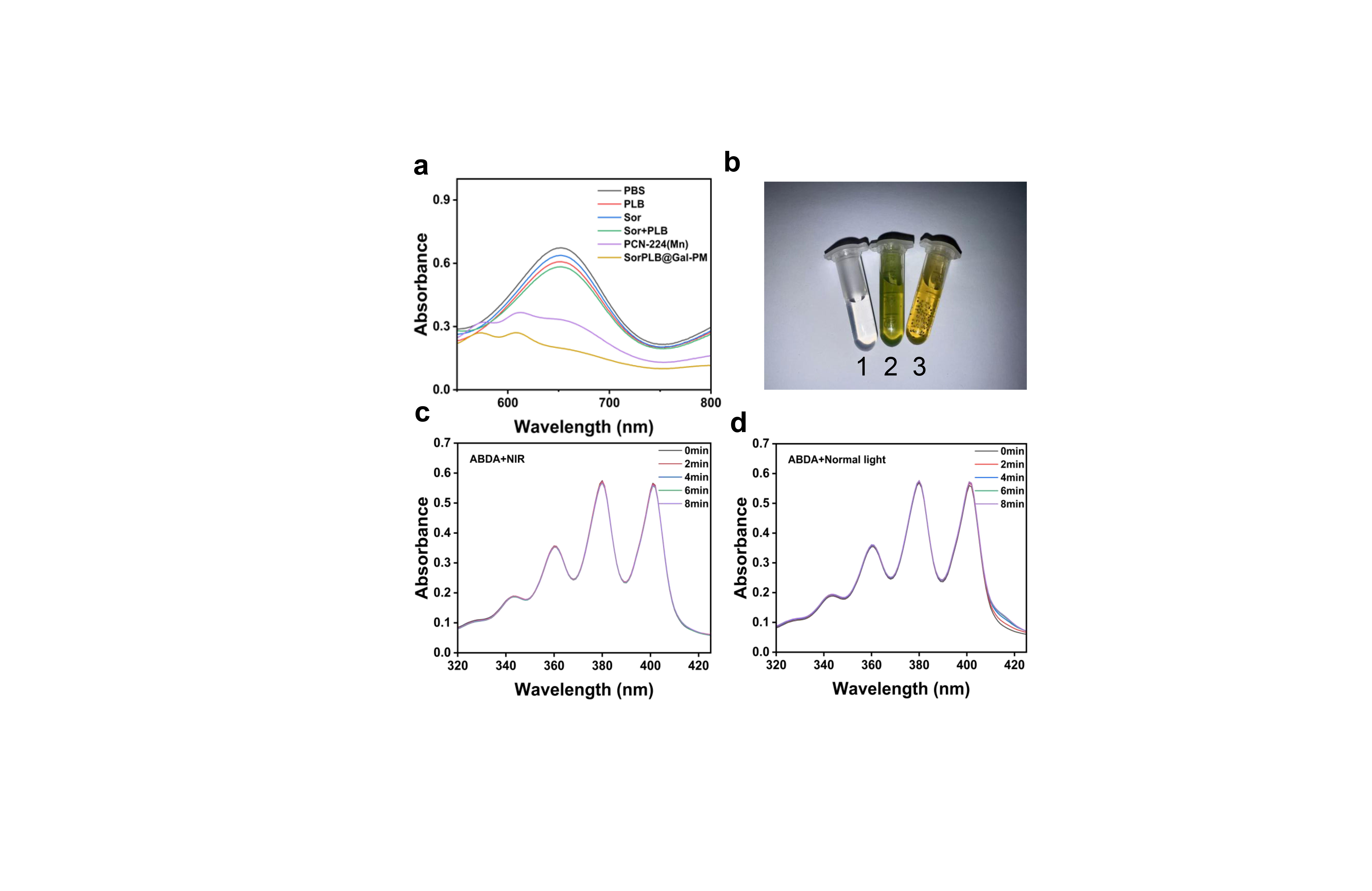


**Fig S2.** (a) UV-vis absorption spectra of TMB/HRP and H_2_O_2_ mixed different treatments after 25 min. (b) Photograph of the H_2_O_2_ solution incubated with: (1) PBS, (2) SorPLB@Gal-PCN-224, (3) SorPLB@Gal-PM. (c) UV-vis spectra of ABDA irradiated with NIR. (d) UV-vis spectra of ABDA irradiated with normal light.


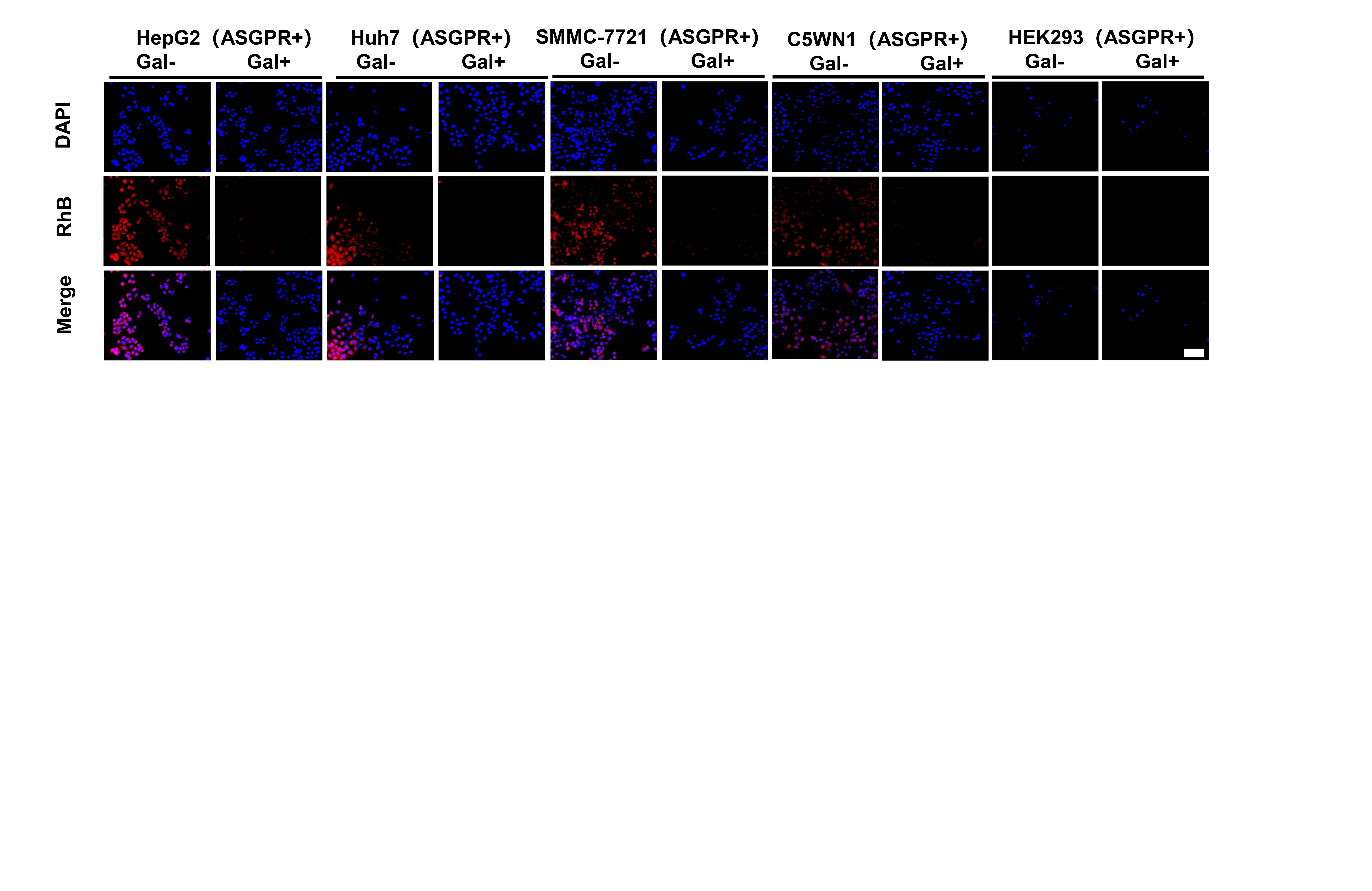


**Fig S3.** Fluorescence microscope images of HepG2, Huh7, SMMC-7721, C5WN1 and HEK293 cells incubated with SorPLB@Gal-PM with or without galactose (1 mM) competition. Scale bar: 100 μm.


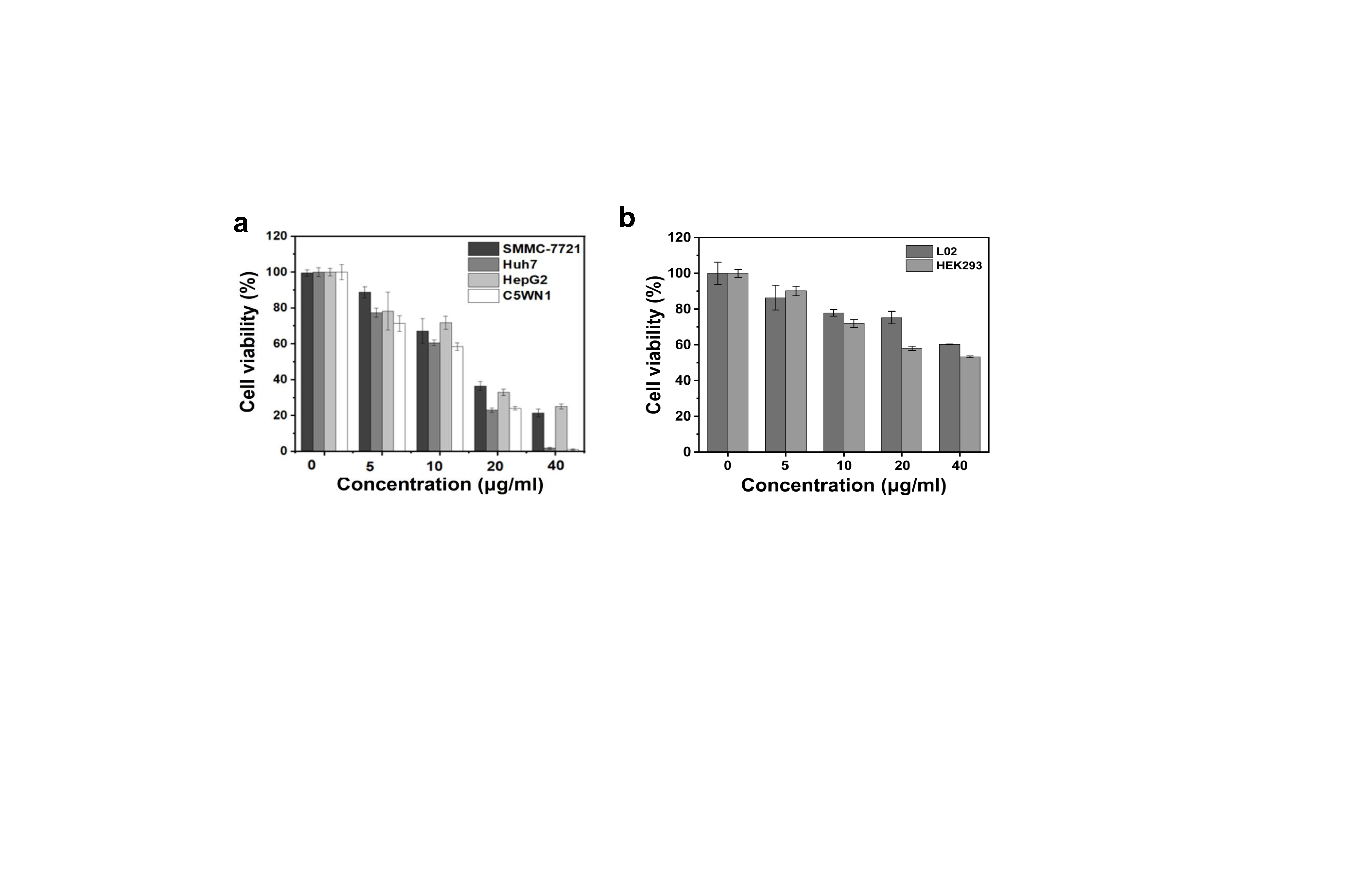


**Fig S4.** (a) Cell viability of SMMC-7721, Huh7, HepG2 and C5WN1 cells incubated with SorPLB@Gal-PM at various concentrations for 48 h. Mean ± SD (n = 3). (b) Cell viability of L02 and HEK293 cells incubated with SorPLB@Gal-PM at various concentrations for 48 h. Mean ± SD (n = 3).

**Table S1. In vitro synergy experiment Inhibition rate**

| **Cell line** | **Inhibition rate of SorPLB@Gal-PM** (*I*_A_) | **Inhibition rate of PM + NIR** (*I*_B_) | **Inhibition rate of SorPLB@Gal-PM + NIR** (*I*_obs_) | ΔI |
| --- | --- | --- | --- | --- |
| SMMC-7721 | 59.78% | 20.01% | 71.09% | 3.25% |
| HepG2 | 61.85% | 22.02% | 74.94% | 4.71% |
| HuH7 | 62.47% | 12.21% | 82.91% | 15.87% |
| C5WN1 | 74.19% | 5.54% | 88.91% | 13.28% |

Note: ΔI =*I*_A_ + *I*_B_-(*I*_A_×*I*_B_)- *I*_obs_





**Fig S5.** Mean fluorescent intensity of DCFH-DA in C5WN1 cells after different treatments.


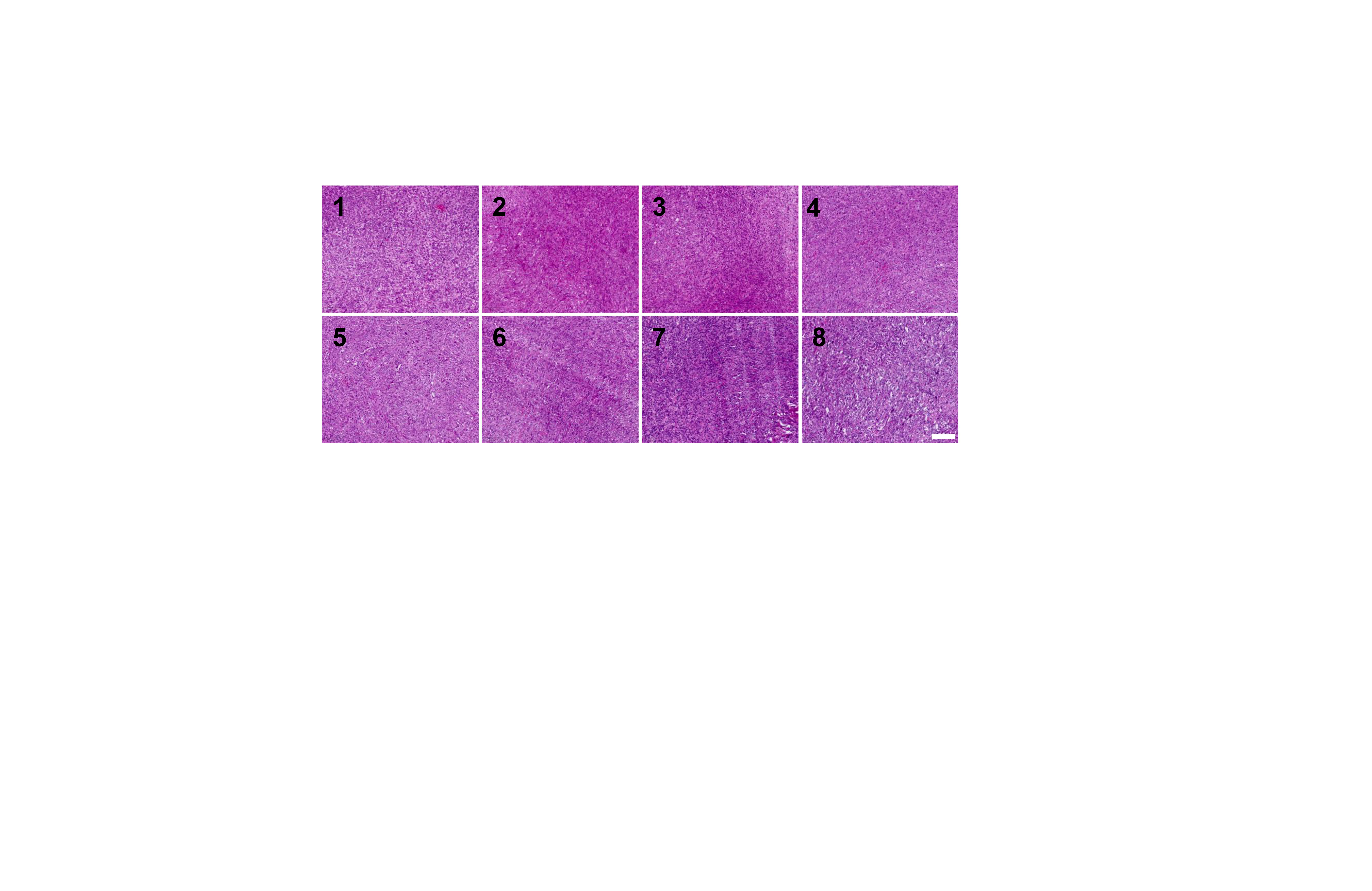


**Fig S6.** H&E staining of tumor tissues in subcutaneous C5WN1-bearing mice after the 14-day treatment. (1) saline, (2) PM, (3) PM + NIR, (4) PLB, (5) Sor + PLB, (6) SorPLB@PM, (7) SorPLB@Gal-PM, (8) SorPLB@Gal-PM + NIR. Scale bar: 100 μm.


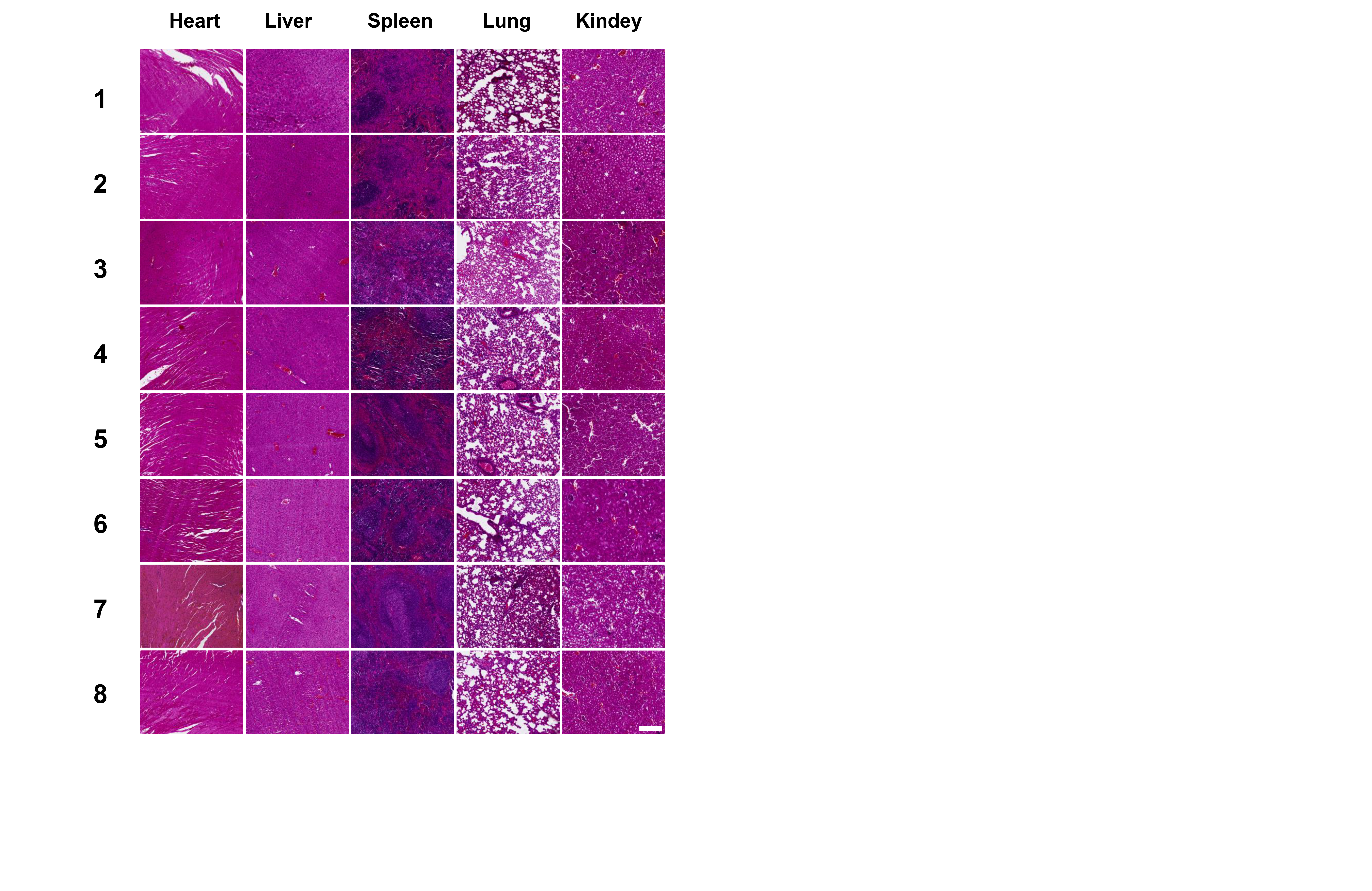


**Fig S7.** H&E staining of organs in subcutaneous C5WN1-bearing mice after the 14-day treatment. (1) saline, (2) PM, (3) PM + NIR, (4) PLB, (5) Sor + PLB, (6) SorPLB@PM, (7) SorPLB@Gal-PM, (8) SorPLB@Gal-PM + NIR. Scale bar: 100 μm.


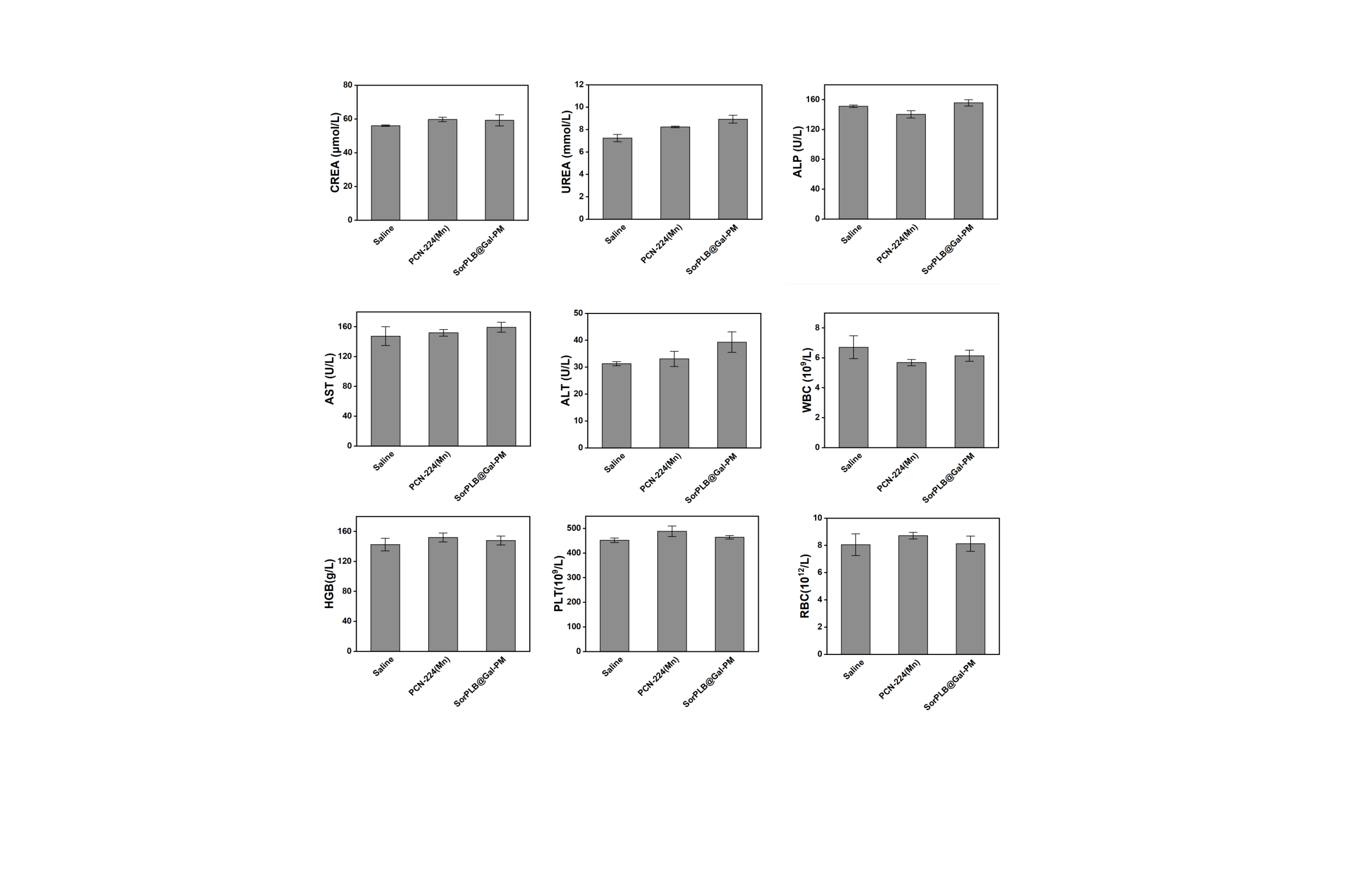


**Fig. S8.** The changes of blood routine and biochemical indexes of C5WN1-bearing mice in different groups after the 14-day treatment. Mean ± SD (n = 3).
